# Antimicrobial Resistance Profile of *Staphylococcus pseudintermedius* Isolated from Dogs with Otitis Externa and Healthy Dogs: Veterinary and Zoonotic Implications

**DOI:** 10.3390/antibiotics14101027

**Published:** 2025-10-14

**Authors:** Ionela Popa, Ionica Iancu, Vlad Iorgoni, Janos Degi, Alexandru Gligor, Kalman Imre, Emil Tîrziu, Timea Bochiș, Călin Pop, Ana-Maria Plotuna, Paula Nistor, Marius Pentea, Viorel Herman, Ileana Nichita

**Affiliations:** 1 Department of Semiology, Faculty of Veterinary Medicine, University of Life Sciences “King Mihai I”, 300645 Timişoara, Romania; ionela.popa@usvt.ro (I.P.); timea.bochis@usvt.ro (T.B.); calinpop@usvt.ro (C.P.); 2 Department of Infectious Diseases and Preventive Medicine, Faculty of Veterinary Medicine, University of Life Sciences “King Mihai I”, 300645 Timisoara, Romania; vlad.iorgoni@usvt.ro (V.I.); janosdegi@usvt.ro (J.D.); alexandru.gligor@usvt.ro (A.G.); paula.nistor@usvt.ro (P.N.); viorel.herman@fmvt.ro (V.H.); 3 Department of Food Safety and Hygiene, Faculty of Veterinary Medicine, University of Life Sciences “King Mihai I”, 300645 Timisoara, Romania; kalmanimre@usvt.ro; 4 Department of Microbiology, Faculty of Veterinary Medicine, University of Life Sciences “King Mihai I”, 300645 Timisoara, Romania; emiltirziu@usvt.ro (E.T.);; 5 Department of Animal Nutrition, University of Life Sciences “King Mihai I”, 300645 Timisoara, Romania; anamaria.plotuna@usvt.ro; 6 Department of Anatomy, University of Life Sciences “King Mihai I”, 300645 Timisoara, Romania; mariuspentea@usvt.ro

**Keywords:** *Staphylococcus pseudintermedius*, antimicrobial resistance, MRSP, otitis externa, dogs, MDR, zoonosis

## Abstract

**Background/Objectives**: *Staphylococcus pseudintermedius* (*S. pseudintermedius*) is an opportunistic pathogen frequently isolated from dogs, involved in a wide range of infections, particularly otitis externa. Increasing antimicrobial resistance (AMR), including methicillin-resistant *S. pseudintermedius* (MRSP), poses significant challenges for veterinary and potentially human health. This study aimed to assess the prevalence and antimicrobial resistance profiles of *S. pseudintermedius* in dogs with otitis externa compared to clinically healthy dogs. **Methods**: Between 2022 and 2025, samples were collected from 400 dogs with otitis externa and 360 healthy dogs in veterinary clinics from Timișoara. Ear swabs were processed by conventional microbiological techniques and confirmed using MALDI-TOF MS. Antimicrobial susceptibility was tested using the VITEK^®^ 2 Compact system, following CLSI VET01, Fifth Edition (2018) standards. Fourteen antimicrobials from 11 classes were evaluated. **Results**: *S. pseudintermedius* was isolated in 40% of dogs with otitis externa and in 21.1% of healthy dogs. The highest resistance in both groups was observed to tetracycline (37.5% and 25%, respectively). No resistance was recorded to linezolid, vancomycin, teicoplanin, tigecycline, or fusidic acid. MRSP strains were identified in 1.2% of dogs with otitis, displaying multidrug resistance (MDR). MDR strains were also detected in 8.7% of diseased and 4% of healthy dogs, indicating the potential for subclinical reservoirs. **Conclusions**: The findings highlight the notable prevalence and AMR of *S. pseudintermedius* in both symptomatic and asymptomatic dogs. The detection of MRSP and MDR strains emphasizes the need for prudent antibiotic use and continuous AMR surveillance in veterinary medicine to mitigate zoonotic risks and preserve antimicrobial efficacy.

## 1. Introduction

*S. pseudintermedius*, an opportunistic pathogen, is commonly, isolated from clinically healthy dogs and is notably involved in a wide spectrum of animal infections [1]. This bacterium can colonize between 46% and 92% of healthy dogs [2]. *S. pseudintermedius* is recognized as a key pathogen in dogs, has also occasionally been linked to cases of human infection [3,4], while it also plays a significant role in infections among cats [4].

The growing identification of *S. pseudintermedius* isolated from canine cases of otitis externa and soft tissue or skin infections represents a rising concern in the veterinary field, primarily due to its considerable resistance to antimicrobials and a broad range of virulence factors [5]. Antimicrobial resistance (AMR) has become a major global health issue, widely recognized as one of the most critical threats of this century, with implications for both human and animal health [6,7,8,9]. In recent years, there has been an observed increase in the frequency of methicillin-resistant *S. pseudintermedius* (MRSP) strains, both in sick dogs and in clinically healthy ones [10]. The spread of MRSP has come to represent a major issue in veterinary practice [11].

MRSP resistance is determined by the presence of the mecA gene, integrated into a mobile genetic element known as the staphylococcal chromosomal cassette mec (SCCmec). This gene confers resistance to most beta-lactam antibiotics, except for ceftaroline and ceftobiprole, which are part of the fifth generation of cephalosporins [11,12].

The mecA gene encodes a structurally altered penicillin-binding protein with low affinity for nearly all beta-lactam antibiotics [10,13]. Consequently, these antibiotics no longer represent a barrier to bacterial cell wall synthesis, unlike under normal circumstances when beta-lactams bind to the penicillin-binding protein and thereby inhibit the bacterium’s ability to construct its cell wall [13]. In addition, the SCCmec acquired by MRSP is a mobile genetic element [10,11,12,13] that may also carry other resistance genes, contributing, along with other mobile genetic elements, to multidrug resistance patterns (MDR) [12]. Resistance of MRSP strains has also often been observed within other classes of antimicrobials, namely: aminoglycosides, fluoroquinolones, macrolides [11,12], trimethoprim–sulfamethoxazole, lincosamides, tetracyclines, and chloramphenicol [12].

*S. pseudintermedius* can be transmitted to humans, particularly those in close contact with dogs, potentially causing zoonotic infections such as abscesses, infected bite wounds, pneumonia, bloodstream infections, and septic arthritis [10]. Consequently, MRSP has become an increasingly significant concern in veterinary as well as human medicine, due to the limited treatment options available [14].

The objectives of this study were to determine the prevalence of *S. pseudintermedius* in dogs with otitis externa compared to clinically healthy dogs, to characterize the antimicrobial resistance profiles of the isolates against both veterinary and human-use antibiotics, to identify MRSP and MDR strains, and to assess their potential zoonotic implications. These aims were designed to provide a clearer understanding of the epidemiological role of *S. pseudintermedius* in canine populations and to support strategies for prudent antimicrobial use in veterinary medicine.

## 2. Results

### 2.1. Prevalence of S. pseudintermedius Isolated from Dogs with Otitis Externa and from the Ears of Healthy Dogs

Out of 400 samples collected from dogs diagnosed with otitis externa, *S. pseudintermedius* was isolated in 160 cases, representing a prevalence rate of 40%. From the ears of clinically healthy dogs the bacterium was identified in 76 out of 360 samples, corresponding to a prevalence of 21.1%.

### 2.2. Antimicrobial Resistance of S. pseudintermedius Strains Isolated from Dogs with Otitis Externa

Among dogs with otitis externa, *S. pseudintermedius* showed the highest resistance to tetracycline (37.5%, *n* = 60). No resistance was observed to linezolid, teicoplanin, vancomycin, fusidic acid, and tigecycline, with 100% susceptibility (*n* = 160) (Table 1).

### 2.3. Antimicrobial Resistance of S. pseudintermedius Strains Isolated from the Ears of Healthy Dogs

Additionally, *S. pseudintermedius* isolated from samples collected from the ears of healthy dogs exhibited the highest resistance to tetracycline as well, at a rate of 25% (*n* = 19) (Table 2).

Analysis by antimicrobial class revealed that the most frequent resistance in both groups occurred within the tetracycline class. In isolates from dogs with otitis externa, 37.5% were resistant to tetracyclines, 23.1% to β-lactams, and 21.9% to lincosamides. In isolates from the ears of healthy dogs, the corresponding resistance rates were 25%, 19.7%, and 22.4%, respectively. All isolates were fully susceptible to glycopeptides, oxazolidinones, fusidanes, and glycylcyclines (Table 2).

### 2.4. Distribution of MDR and MRSP Strains

Regarding MDR, 14 strains (8.7%) were isolated from samples collected from dogs with otitis externa (Table 3), while 3 strains (4%) were isolated from samples taken from the ears of healthy dogs (Table 4).

Additionally, two MRSP strains (1.2%) were isolated from samples collected from dogs with otitis externa (Table 3).

Regarding the MRSP strains, in addition to resistance to β-lactam antibiotics, they also exhibited resistance to antimicrobials from the following classes: aminoglycosides, tetracyclines, fluoroquinolones, macrolides, lincosamides, and sulfonamides + pyrimidines (Table 3).

A comparative statistical analysis of *S. pseudintermedius* prevalence and antimicrobial resistance between isolates from dogs with otitis externa and those from clinically healthy dogs is presented below (Table 5). This analysis includes *p*-values, false discovery rate (FDR)-adjusted *q*-values, risk differences (RD), and relative risks (RR) with 95% confidence intervals, providing an inferential assessment of the significance and magnitude of the observed differences.

## 3. Discussion

The prevalence of *S. pseudintermedius* in this study was 40%, which is consistent with findings reported by Hassan et al. [15] (41.6%) and Penna et al. [16] (38.4%), and somewhat higher than the 31.5% reported by De Martino et al. [17]. These similarities may reflect broadly comparable epidemiological conditions and sampling strategies across studies, such as targeting clinical isolates from companion animals with similar clinical presentations. However, slight differences in prevalence could stem from geographic variability, including differences in population density, pet ownership practices, and local veterinary diagnostic capacities. Moreover, differences in study design, such as sample size or inclusion criteria, could also influence prevalence rates and partially account for the variability observed across studies.

AMR profile revealed a predominance of tetracycline resistance (37.5%) (Table 1), consistent with De Martino et al. [17] (35.5%) and Rosales et al. [18] (41.7%). The slightly lower or higher values observed in these studies likely reflect variations in local antimicrobial usage patterns. For example, tetracyclines remain widely used in veterinary medicine due to their broad-spectrum activity and affordability, which may contribute to a sustained selective pressure favoring resistant strains. The even higher resistance rate reported by Tesin et al. [19] (52%) may be influenced by regional overuse or misuse of tetracyclines, as well as the inclusion of isolates from animals with recurrent infections, where resistance is typically higher.

Resistance to penicillin was observed in 23.1% of isolates. This rate is considerably lower than the values reported by Bourély et al. [20] (68.5%), Rosales et al. [18] (69%), and Scherer et al. [21] (77.3%), but higher than the 7% reported by Rubin et al. [22]. Several factors may account for these discrepancies. First, methodological differences, such as the antimicrobial susceptibility testing method employed (e.g., disk diffusion vs. MIC determination), the inclusion or exclusion of intermediate isolates, and the interpretive criteria applied (e.g., CLSI vs. CA-SFM), can significantly influence reported resistance rates [22]. Second, the studies differ in the bacterial populations analyzed: while our study focused exclusively on *S. pseudintermedius*, others may have included mixed staphylococcal species or isolates pre-selected based on methicillin susceptibility, which could introduce selection bias. Third, regional variation in antimicrobial stewardship strategies, veterinary prescribing behaviors, and regulatory frameworks likely contribute to differences in resistance patterns. Given these substantial methodological and epidemiological differences, direct comparisons across studies should be interpreted with caution.

Clindamycin resistance was observed in 21.9% of isolates, aligning with Rosales et al. [18] (29.4%) but higher than the 9% reported by Norström et al. [23]. These differences may reflect variation in the therapeutic use of lincosamides across regions. Moreover, Norström et al. [23] isolated *S. pseudintermedius* from both otic and skin infections, and it is possible that site-specific differences in bacterial populations or exposure to clindamycin contributed to the lower resistance observed.

Gentamicin resistance was low (1.3%) in our study, which matches the 1% reported by Bugden [24] and is notably lower than values from De Martino et al. [17] (11.1%), Bourély et al. [20] (13.5%), and Rosales et al. [18] (17.6%). This finding may suggest that aminoglycosides are either used sparingly or mainly in severe infections where culture and sensitivity testing are performed, limiting their contribution to resistance selection.

Similarly, erythromycin resistance was very limited (1.3%), in stark contrast to the much higher rates reported by Rosales et al. [18] (29.7%), Bourély et al. [20] (29.8%), and especially Penna et al. [16] (80%). These discrepancies likely reflect regional differences in antimicrobial policies and prescription behaviors, and possibly different exposure histories of the bacterial populations sampled.

Trimethoprim-sulfamethoxazole resistance was found in only 3.8% of isolates, consistent with Rubin et al. [22] (5%), but considerably lower than rates reported by Rosales et al. [18] (18%) and De Martino et al. [17] (46.6%). The low resistance rate observed in our study may indicate either limited use of this antibiotic in clinical veterinary practice or its continued efficacy due to stewardship efforts. It is also possible that local veterinary guidelines prioritize other antimicrobials, thus reducing the selective pressure for resistance to this compound.

In the present study, the susceptibility of *S. pseudintermedius* isolates was assessed with respect to several antimicrobials reserved solely for human use. These included linezolid, vancomycin, tigecycline, and teicoplanin—agents classified as important and reserved for the treatment of human infections. Additionally, moxifloxacin, a fluoroquinolone approved for human use, was tested [25]. All isolates were susceptible to antibiotics reserved for human use (linezolid, vancomycin, tigecycline, and teicoplanin), demonstrating 100% susceptibility (*n* = 160) (Table 1 and Table 2). In contrast, a resistance rate of 1.3% (*n* = 2) was observed for moxifloxacin (Table 1). The monitoring of AMR should extend beyond agents exclusively used in veterinary medicine, given the zoonotic threat posed by MRSP and its capacity to spread AMR genes via horizontal gene transfer [26].

Regarding MDR strains, they were isolated at a rate of 8.7% in this study (Table 3). Our findings differ from those reported by Viegas et al. [11], who documented an MDR prevalence of 14.5% among *S. pseudintermedius* strains isolated from canine external otitis cases.

The proliferation MDR bacterial strains constitutes a critical threat to global health, as underscored by the World Health Organization. The rising incidence of infections attributable to MDR organisms, alongside the dwindling efficacy of current therapeutic options, is projected to contribute to increased fatality levels among animal and human hosts affected by such diseases. MDR is generally defined by resistance to antimicrobial agents spanning a minimum of three distinct antimicrobial classes [27].

Regarding the MRSP strains, they were isolated at a rate of 1.2% in this study (Table 3). Our results differ from those reported by Viegas et al. [11], who found MRSP strains in 17.6% of dogs with external otitis. According to the literature, the prevalence of MRSP among canine populations shows wide variation, with reported values spanning from 0% to 60% [11]. It is important to note that drawing direct comparisons across studies can be challenging, as MRSP prevalence is influenced by multiple factors, including the study population, sample type, geographic location, and methodological approach [11].

According to CLSI guidelines, staphylococci showing resistance to oxacillin are interpreted as resistant to each class of β-lactam antibiotics. In antimicrobial susceptibility testing, methicillin was substituted with oxacillin due to its greater stability [28]. Beyond their resistance to β-lactam antibiotics, MRSP strains commonly possess resistance to several other antimicrobial drug classes [13].

Regarding penicillin resistance among *S. pseudintermedius* strains isolated from the ears of healthy dogs, the prevalence observed in this study was 19.7% (Table 2). This value is lower than the 39.9% reported by Rubin and Chirino-Trejo [29], who analyzed isolates from nasal, pharyngeal, and rectal sites. One possible explanation for this discrepancy is the anatomical site of sampling, as microbial populations and resistance profiles can vary significantly between different body regions due to differences in local microenvironments and antimicrobial exposure. In addition, geographic variation and differences in antimicrobial use practices between populations or regions may also contribute to this divergence.

Similarly, the tetracycline resistance rate in our study was 25%, closely aligning with the 23.5% reported by Rubin and Chirino-Trejo [29]. This consistency might suggest a more stable pattern of tetracycline resistance across different body sites and potentially across different geographical regions. However, the similarity could also reflect the widespread and long-term use of tetracyclines in veterinary medicine, which may have exerted consistent selective pressure over time.

Regarding MRSP, no strains were identified in our study. This is consistent with the findings of Rubin and Chirino-Trejo [29], who also reported no MRSP in clinically healthy dogs. The absence of MRSP in both studies could indicate a relatively low prevalence of methicillin resistance in *S. pseudintermedius* among healthy canine populations, at least in the sampled regions. It may also suggest that MRSP carriage is more closely associated with clinical infections [30] or prior antimicrobial exposure, which were not present in the healthy dogs studied.

The current understanding of the epidemiology, zoonotic potential, and antimicrobial resistance patterns of *S. pseudintermedius* in healthy canine carriers remains limited. Given the implications for both veterinary and public health, further investigations are essential to better characterize and address these critical areas [29].

In this study, the *S. pseudintermedius* strains isolated from dogs with otitis externa exhibited a more diverse and extensive antimicrobial resistance profile compared to those isolated from clinically healthy dogs. Although the proportion of strains susceptible to all tested antimicrobials was relatively similar between the two groups (38.1% in dogs with otitis externa vs. 40.8% in healthy dogs), resistance to tetracycline, penicillin, and clindamycin was more frequently observed among pathogenic strains. Notably, MRSP strains were exclusively isolated from dogs with otitis externa. All isolates were susceptible to antibiotics reserved for human medicine—such as linezolid, teicoplanin, vancomycin, and tigecycline—which were included in the testing panel due to the zoonotic potential of *S. pseudintermedius*. No resistance was observed to fusidic acid either. These findings support the notion that *S. pseudintermedius* strains isolated from both healthy and diseased dogs may harbor antimicrobial resistance and pose potential risks for zoonotic transmission, although strains associated with otitis externa tend to exhibit more frequent and complex resistance patterns.

Recent research has shown that *S. pseudintermedius*, particularly methicillin-resistant strains (MRSP), may pose a zoonotic risk. Sequence types such as ST45, ST71, and ST121 were detected in both companion animals and human clinical samples, and whole-genome comparisons revealed high similarity between isolates, suggesting possible recent interspecies transmission [31].

Genomic analysis revealed notable diversity, including 23 STs among animal-derived strains and ten novel variants. Most isolates carried common resistance genes like *mecA* and *blaZ*, along with others conferring resistance to aminoglycosides, macrolides, and tetracyclines. Virulence factors such as *sps*, *ica*, and *leukotoxin* genes were also present and appeared to vary by sequence type, suggesting host adaptation [31].

While no direct correlation between host and ST was found, the genetic overlap between isolates from animals and humans highlights the zoonotic potential of MRSP and supports the need for One Health surveillance strategies [31].

Mobile genetic elements, such as SCCmec types III, IVg, V, and VII, were identified across MRSP isolates and played a key role in the dissemination of resistance genes, particularly *mecA*. Specific point mutations associated with fluoroquinolone resistance (e.g., grlA S80I and gyrA S84L) were frequently observed, especially in ST71 and ST339. These genomic features, alongside the presence of biocide resistance and virulence-associated genes, underline the adaptability of *S. pseudintermedius* and its potential to persist in both veterinary and human environments [32].

Moreover, the recent literature provides additional context that complements our findings. Regional studies have highlighted the clinical relevance of *S. pseudintermedius* and other staphylococcal species isolated from cutaneous and mucosal infections in companion animals [33,34]. Comprehensive reviews have described major epidemiological shifts in MRSP prevalence and explored alternative therapeutic strategies to address emerging resistance patterns [35]. Large-scale surveillance investigations from European veterinary clinics have further reported substantial MDR prevalence and complex co-resistance profiles among *S. pseudintermedius* isolates [36]. In addition, meta-analyses of canine otitis externa and pyoderma cases confirm a wide range of prevalence values across geographical regions [37], while recent studies emphasize the influence of host-related and management-associated factors on the development and spread of antimicrobial resistance in canine populations [38].

The statistical analysis further reinforces the robustness of our findings, demonstrating significant differences in prevalence and antimicrobial resistance between isolates from diseased and clinically healthy dogs. The inclusion of *p*-values, FDR-adjusted *q*-values, and relative risk estimates provides quantitative evidence of these differences, underscoring their clinical and epidemiological relevance.

### 3.1. Limitations

This study has some limitations. The sampling was restricted to a single urban area, which may affect the generalizability of the findings. Furthermore, genetic characterization of resistance determinants was not performed.

### 3.2. Strengths

By documenting the presence of methicillin-resistant and multidrug-resistant *S. pseudintermedius* in dogs, this study highlights the zoonotic risks and reinforces the importance of prudent antimicrobial use within a One Health perspective.

## 4. Materials and Methods

### 4.1. Study Design

The study was conducted between 2022 and 2025 and involved the collection of samples from dogs diagnosed with otitis externa, as well as from clinically healthy dogs presented for routine procedures (such as sterilization, vaccination, or deworming) at various veterinary clinics in Timișoara, Romania.

### 4.2. Sample Collection

Samples were collected from 400 dogs presenting clinical signs of otitis externa and from 360 clinically healthy dogs. Bilateral ear swabs were taken using sterile cotton swabs, which were immediately placed in Amies (Oxoid, Basingstoke, UK) transport medium and stored at 4 °C for a maximum of 24 h prior to processing.

Samples were collected from 400 dogs presenting clinical signs of otitis externa and 360 clinically healthy dogs. Bilateral ear swabs were obtained using sterile cotton swabs, placed in Amies transport medium, and stored at 4 °C for a maximum of 24 h prior to processing. Although samples were collected from both ears, only one *S*. *pseudintermedius* isolate per dog was included in the final analysis. This methodological choice was made to prevent pseudoreplication and overrepresentation of clonal isolates from the same individual, which could bias prevalence and antimicrobial resistance estimations. By focusing on a single representative isolate per host, the study ensured that epidemiological comparisons between diseased and healthy dogs reflected true host-level dynamics rather than intra-individual variability.

In cases where multiple *S*. *pseudintermedius* isolates displaying identical antimicrobial resistance profiles were recovered from the same animal, only one representative isolate was retained to avoid data duplication and potential overestimation of strain prevalence. While this approach may have limited the detection of intra-host diversity and potential strain coexistence, it provided a more robust and accurate assessment of *S*. *pseudintermedius* occurrence and resistance patterns across the study population. This strategy aligns with standard epidemiological practices for prevalence and resistance studies, emphasizing comparability between clinical and healthy cohorts.

We acknowledge that phenotypic similarity does not necessarily imply clonal identity, as horizontal gene transfer or convergent evolution may result in similar resistance phenotypes among distinct strains. Therefore, future research incorporating molecular typing techniques, such as multilocus sequence typing (MLST), pulsed-field gel electrophoresis (PFGE), or whole-genome sequencing (WGS), is warranted to explore intra-host diversity and confirm genetic relatedness among *S*. *pseudintermedius* isolates.

### 4.3. Bacterial Isolation and Species Identification

Ear swab samples were cultured on two types of media: Columbia agar supplemented with 5% sheep blood ((Oxoid, Basingstoke, UK), a non-selective enriched medium that allows hemolysis evaluation, and Chapman agar (Mannitol Salt Agar; bioMérieux, Marcy-l’Étoile, France), a selective and differential medium for the isolation of halotolerant staphylococci and their differentiation based on mannitol fermentation. The plates were incubated aerobically at 35 °C for 18–24 h. Suspected colonies were selected based on characteristic morphology and subjected to Gram staining, catalase testing, and, when appropriate, coagulase testing. Suspect colonies were subcultured to obtain pure isolates, which were subsequently identified using MALDI-TOF mass spectrometry.

Prior to antimicrobial susceptibility testing, presumptive *S. pseudintermedius* isolates were identified using MALDI-TOF MS (matrix-assisted laser desorption/ionization time-of-flight mass spectrometry; Bruker Daltonik, Bremen, Germany). Bacterial protein extracts were prepared following a standard ethanol/formic acid protocol. A volume of 1 µL from the prepared sample was placed on a MALDI target plate, and subsequently layered with 1 µL of matrix solution composed of α-cyano-4-hydroxycinnamic acid (10 mg/mL), prepared in 50% acetonitrile mixed with 2.5% trifluoroacetic acid. Spectra acquisition was performed using a Microflex™ mass spectrometer (Bruker Daltonik GmbH, Bremen, Germany), and data were processed via MALDI BioTyper™ 3.0 software (Bruker Daltonik GmbH, Bremen, Germany). Species determination was based on spectral comparison against the manufacturer’s database. According to the scoring criteria provided by Bruker, values ≥ 2.0 were accepted as reliable species-level identification, whereas scores ranging from 1.7 to 1.99 were interpreted as indicative of genus-level classification [39].

Quality control (QC) for MALDI-TOF identification was initially performed using *Staphylococcus aureus* ATCC 25923 as a general control strain, in accordance with manufacturer recommendations. To further ensure the accuracy of species identification, a representative subset of isolates was re-analyzed using the well-characterized *S. pseudintermedius* DSM 21284 reference strain as a species-specific QC. The results confirmed the identity of all isolates originally included in the study, thereby reinforcing the robustness of the identification procedure. Moreover, the Bruker Biotyper database used in this study contained manufacturer-validated protein spectra for *S. pseudintermedius*, ensuring accurate and reliable identification.

The Biotyper database used included manufacturer-validated protein spectra for *S. pseudintermedius*.

### 4.4. Antimicrobial Susceptibility Testing (AST)

Antimicrobial susceptibility was assessed with the VITEK^®^ 2 Compact system (bioMérieux, Marcy-l’Étoile, France), in line with the manufacturer’s recommendations. Initially, pure bacterial colonies were isolated, and suspensions were made in sterile saline, with turbidity adjusted to correspond to the 0.5 McFarland standard. To evaluate resistance, specific cards for Gram-positive strains (VITEK® AST-GP79; bioMérieux, Marcy-l’Étoile, France) were employed [40].

Antimicrobial susceptibility profiling was performed using 14 antimicrobial agents belonging to 11 different classes. The tested substances included: penicillin and oxacillin (β-lactams); gentamicin (aminoglycosides); tetracycline (tetracyclines); ciprofloxacin and moxifloxacin (fluoroquinolones); erythromycin (macrolides); clindamycin (lincosamides); linezolid (oxazolidinones); teicoplanin and vancomycin (glycopeptides); fusidic acid (fusidanes); tigecycline (glycylcyclines); and trimethoprim–sulfamethoxazole (sulfonamides + pyrimidines).

Calibration and quality control of the VITEK^®^ 2 Compact system were performed daily according to the manufacturer’s instructions, using the quality control strain *Staphylococcus aureus* ATCC 29213.

Interpretation of susceptibility results was carried out in accordance with the CLSI VET01, Fifth Edition (2018) standard, which is specifically designed for bacterial isolates from animals [41].

### 4.5. Ethical Approval

Ethical approval for this study was granted by the Bioethics Commission of the University of Life Sciences “King Mihai I” in Timișoara (Approval No. 592, dated 9 September 2025). The research was conducted without involving experimental procedures on live animals and complied with all relevant ethical standards for animal research.

Statistical analyses were conducted using Chi-square or Fisher’s exact tests for proportions, with significance set at *p* < 0.05; false discovery rate (FDR) adjustment, risk difference (RD), and relative risk (RR) with 95% confidence intervals were reported.

## 5. Conclusions

*S. pseudintermedius* was identified with a significant prevalence in both dogs with otitis externa (40%) and clinically healthy dogs (21.1%), highlighting its role as a relevant pathogen and a potential community reservoir. The antimicrobial resistance profile revealed a notably high resistance to tetracyclines in both groups, indicating a selective pressure associated with the frequent use of this antibiotic class.

MRSP was detected at a low rate (1.2%) among dogs with otitis externa; however, MRSP strains exhibited multidrug resistance, including resistance to other critically important antimicrobial classes. This raises concerns regarding the therapeutic challenges posed by these infections and the potential for dissemination of resistant strains.

Moreover, MDR isolates were present in both diseased and healthy dogs, suggesting that clinically healthy animals may act as important reservoirs of resistant bacteria with potential zoonotic implications.

These findings underscore the urgent need for robust antimicrobial resistance surveillance strategies and the prudent use of antibiotics in veterinary medicine, in order to limit the spread of resistant strains and safeguard both animal and public health.

## Figures and Tables

**Table 1:** Antimicrobial resistance of *S. pseudintermedius* strains isolated from dogs with otitis externa.

Antimicrobial Class	Antimicrobial	Number of Strains Tested	Susceptible, *n* (%)	Resistant, *n* (%)
Beta-lactams	Penicillin	160	123 (80.3)	37 (19.7)
	Oxacillin	160	158 (98.7)	2 (1.3)
Aminoglycosides	Gentamicin	160	158 (98.7)	2 (1.3)
Tetracyclines	Tetracycline	160	100 (62.5)	60 (37.5)
Fluoroquinolones	Ciprofloxacin	160	158 (98.7)	2 (1.3)
	Moxifloxacin	160	158 (98.7)	2 (1.3)
Macrolides	Erythromycin	160	158 (98.7)	2 (1.3)
Lincosamides	Clindamycin	160	125 (78.1)	35 (21.9)
Oxazolidinones	Linezolid	160	160 (100)	0 (0)
Glycopeptides	Teicoplanin	160	160 (100)	0 (0)
	Vancomycin	160	160 (100)	0 (0)
Fusidans	Fusidic acid	160	160 (100)	0 (0)
Glycylcyclines	Tigecycline	160	160 (100)	0 (0)
Sulfonamides + Pyrimidines	Trimethoprim + Sulfamethoxazole	160	154 (96.2)	6 (3.8)

**Table 2:** Antimicrobial resistance of *S. pseudintermedius* strains isolated from the ears of healthy dogs.

Antimicrobial Class	Antimicrobial	Number of Strains Tested	Susceptible, *n* (%)	Resistant, *n* (%)
Beta-lactams	Penicillin	76	61 (80.3)	15 (19.7)
	Oxacillin	76	76 (100)	0 (0)
Aminoglycosides	Gentamicin	76	76 (100)	0 (0)
Tetracyclines	Tetracycline	76	57 (75)	19 (25)
Fluoroquinolones	Ciprofloxacin	76	76 (100)	0 (0)
	Moxifloxacin	76	76 (100)	0 (0)
Macrolides	Erythromycin	76	76 (100)	0 (0)
Lincosamides	Clindamycin	76	59 (77.6)	17 (22.4)
Oxazolidinones	Linezolid	76	76 (100)	0 (0)
Glycopeptides	Teicoplanin	76	76 (100)	0 (0)
	Vancomycin	76	76 (100)	0 (0)
Fusidans	Fusidic acid	76	76 (100)	0 (0)
Glycylcyclines	Tigecycline	76	76 (100)	0 (0)
Sulfonamides + Pyrimidines	Trimethoprim + Sulfamethoxazole	76	76 (100)	0 (0)

**Table 3:** Antimicrobial resistance profiles of *S. pseudintermedius* strains isolated from dogs with otitis externa (distribution according to the resistance profile of each strain).

Antimicrobial Resistance	Number of Strains and Percentage
Susceptible to all tested antimicrobials	61 (38.1%)
PEN	14 (8.8%)
TET	37 (23.1%)
CLI	25 (15.6%)
TET + PEN	9 (5.6%)
PEN + TET + CLI	8 (5%)
PEN + TET + SXT	4 (2.5%)
PEN + OXA + GEN + TET + CIP + MXF + ERY + CLI + SXT	2 (1.2%)

Legend: PEN—penicillin; OXA—oxacillin; GEN—gentamicin; TET—tetracycline; CIP—ciprofloxacin; MXF—moxifloxacin; ERY—erythromycin; CLI—clindamycin; SXT—trimethoprim + sulfamethoxazole.

**Table 4:** Antimicrobial resistance of *S. pseudintermedius* strains isolated from the ears of healthy dogs (distribution based on resistance profile of each isolate).

Antimicrobial Resistance	Number of Strains and Percentage
Susceptible to all tested antimicrobials	31 (40.8%)
CLI	14 (18.4%)
TET	16 (21.1%)
PEN	12 (15.8%)
PEN + TET + CLI	3 (4%)

Legend: PEN—penicillin; CLI—clindamycin; TET—tetracycline.

**Table 5:** Statistical comparison of antimicrobial resistance rates in *S. pseudintermedius* isolates from dogs with otitis externa and from clinically healthy dogs.

Antimicrobial/Parameter	Otitis Externa (*n*/*N*, %)	Healthy Dogs (*n*/*N*, %)	*p*-Value	*q*-Value (FDR)	Risk Difference (%, 95% CI)	Relative Risk (95% CI)
Prevalence (*S. pseudintermedius*)	160/400 (40.0%)	76/360 (21.1%)	<0.0001	—	18.9% (12.2%, 25.6%)	1.90 (1.52–2.36)
Penicillin resistance	37/160 (23.1%)	15/76 (19.7%)	0.5712	0.6614	3.4% (−8.4%, 15.2%)	1.17 (0.67–2.04)
Oxacillin resistance (MRSP)	2/160 (1.3%)	0/76 (0.0%)	0.5303	0.6614	1.3% (−1.3%, 4.0%)	3.39 (0.17–66.7)
Gentamicin resistance	2/160 (1.3%)	0/76 (0.0%)	0.5303	0.6614	1.3% (−1.3%, 4.0%)	3.39 (0.17–66.7)
Tetracycline resistance	60/160 (37.5%)	19/76 (25.0%)	0.0714	0.1429	12.5% (−1.1%, 26.1%)	1.50 (0.96–2.33)
Ciprofloxacin resistance	2/160 (1.3%)	0/76 (0.0%)	0.5303	0.6614	1.3% (−1.3%, 4.0%)	3.39 (0.17–66.7)
Moxifloxacin resistance	2/160 (1.3%)	0/76 (0.0%)	0.5303	0.6614	1.3% (−1.3%, 4.0%)	3.39 (0.17–66.7)
Erythromycin resistance	2/160 (1.3%)	0/76 (0.0%)	0.5303	0.6614	1.3% (−1.3%, 4.0%)	3.39 (0.17–66.7)
Clindamycin resistance	35/160 (21.9%)	17/76 (22.4%)	0.9284	0.9284	−0.5% (−12.8%, 11.8%)	0.98 (0.58–1.64)
Trimethoprim–Sulfamethoxazole resistance	6/160 (3.8%)	0/76 (0.0%)	0.1832	0.3664	3.8% (−0.9%, 8.4%)	6.15 (0.35–107.8)
MDR (≥3 classes)	14/160 (8.7%)	3/76 (4.0%)	0.2845	0.4735	4.7% (−3.9%, 13.3%)	2.17 (0.64–7.31)
MRSP (oxacillin-resistant)	2/160 (1.3%)	0/76 (0.0%)	0.5303	0.6614	1.3% (–1.3%, 4.0%)	3.39 (0.17–66.7)

Note: *p*-values < 0.05 were considered statistically significant. False discovery rate (FDR)-adjusted *q*-values were calculated using the Benjamini–Hochberg procedure and are presented for comparisons involving multiple antimicrobial resistance outcomes. The prevalence comparison was not included in the multiple testing correction as it represents a single test; therefore, only the raw *p*-value is reported for this parameter. Risk difference (RD) and relative risk (RR) are provided with 95% confidence intervals (CI). RD represents the absolute difference in resistance proportions between the two groups, whereas RR reflects the relative likelihood of resistance in isolates from dogs with otitis externa compared to those from clinically healthy dogs.

## Data Availability

The original contributions presented in this study are included in the article. Further inquiries can be directed to the corresponding author.

## Abbreviations

The following abbreviations are used in this manuscript:

*S. pseudintermedius*	*Staphylococcus pseudintermedius*
MRSP	Methicillin-resistant *Staphylococcus pseudintermedius*
AMR	Antimicrobial resistance
SCCmec	Staphylococcal chromosomal cassette mec
MDR	Multidrug resistance
CLSI	Clinical and Laboratory Standards Institute
MALDI-TOF MS	Matrix-assisted laser desorption/ionization time-of-flight mass spectrometry
BTS	Bacterial Test Standard
AST	Antimicrobial susceptibility testing

## References

[1] Lynch S.A., Helbig K.J. (2021). The Complex Diseases of *Staphylococcus pseudintermedius* in Canines: Where to Next?. Vet. Sci..

[2] Viñes J., Fàbregas N., Pérez D., Cuscó A., Fonticoba R., Francino O., Ferrer L., Migura-Garcia L. (2022). Concordance between Antimicrobial Resistance Phenotype and Genotype of *Staphylococcus pseudintermedius* from Healthy Dogs. Antibiotics.

[3] Wegener A., Broens E.M., Zomer A., Spaninks M., Wagenaar J.A., Duim B. (2018). Comparative genomics of phenotypic antimicrobial resistances in methicillin-resistant *Staphylococcus pseudintermedius* of canine origin. Vet. Microbiol..

[4] Duim B., Verstappen K.M., Broens E.M., Laarhoven L.M., van Duijkeren E., Hordijk J., de Heus P., Spaninks M., Timmerman A.J., Wagenaar J.A. (2016). Changes in the Population of Methicillin-Resistant *Staphylococcus pseudintermedius* and Dissemination of Antimicrobial-Resistant Phenotypes in the Netherlands. J. Clin. Microbiol..

[5] Lee G.Y., Yang S.J. (2020). Comparative assessment of genotypic and phenotypic correlates of *Staphylococcus pseudintermedius* strains isolated from dogs with otitis externa and healthy dogs. Comp. Immunol. Microbiol. Infect. Dis..

[6] Popa I., Imre K., Morar A., Iancu I., Iorgoni V., Bochiș T., Pop C., Gligor A., Florea T., Popa S.A. (2025). Questionnaire-Based Survey Regarding the Rational Usage of Antimicrobial Agents in Food-Producing Animals in Romania. Vet. Sci..

[7] Parasana D.K., Kalyani I.H., Kachchhi A.V., Koringa P.G., Makwana P.M., Patel D.R., Ramani U.V., Javia B.B., Ghodasara S.N. (2025). Profiling of antimicrobial resistance genes from *Staphylococcus pseudintermedius* isolated from dogs with pyoderma using whole genome sequencing. Comp. Immunol. Microbiol. Infect. Dis..

[8] Ertas Onmaz N., Demirezen Yilmaz D., Imre K., Morar A., Gungor C., Yilmaz S., Gundog D.A., Dishan A., Herman V., Gungor G. (2022). Green Synthesis of Gold Nanoflowers Using *Rosmarinus officinalis* and *Helichrysum italicum* Extracts: Comparative Studies of Their Antimicrobial and Antibiofilm Activities. Antibiotics.

[9] Abdallah R., Mostafa N.Y., Kirrella G.A.K., Gaballah I., Imre K., Morar A., Herman V., Sallam K.I., Elshebrawy H.A. (2023). Antimicrobial Effect of Moringa oleifera Leaves Extract on Foodborne Pathogens in Ground Beef. Foods.

[10] Krapf M., Müller E., Reissig A., Slickers P., Braun S.D., Ehricht R., Monecke S. (2019). Molecular characterisation of methicillin-resistant *Staphylococcus pseudintermedius* from dogs and the description of their SCCmec elements. Vet. Microbiol..

[11] Viegas F.M., Santana J.A., Silva B.A., Xavier R.G.C., Bonisson C.T., Câmara J.L.S., Rennó M.C., Cunha J.L.R., Figueiredo H.C.P., Lobato F.C.F. (2022). Occurrence and characterization of methicillin-resistant *Staphylococcus* spp. in diseased dogs in Brazil. PLoS ONE.

[12] Morais C., Costa S.S., Leal M., Ramos B., Andrade M., Ferreira C., Abrantes P., Pomba C., Couto I. (2023). Genetic diversity and antimicrobial resistance profiles of *Staphylococcus pseudintermedius* associated with skin and soft-tissue infections in companion animals in Lisbon, Portugal. Front. Microbiol..

[13] Van Duijkeren E., Catry B., Greko C., Moreno M.A., Pomba M.C., Pyörälä S., Ruzauskas M., Sanders P., Threlfall E.J., Torren-Edo J. (2011). Review on methicillin-resistant *Staphylococcus pseudintermedius*. J. Antimicrob. Chemother..

[14] Moses I.B., Santos F.F., Gales A.C. (2023). Human colonization and infection by *Staphylococcus pseudintermedius*: An emerging and underestimated zoonotic pathogen. Microorganisms.

[15] Hassan M., Kekeç A.I., Halaç B. (2023). Otitis externa in dogs: Distribution and antimicrobial susceptibility patterns of *Staphylococcus* spp. isolates. Mac. Vet. Rev..

[16] Penna B., Varges R., Medeiros L., Martins G.M., Martins R.R., Lilenbaum W. (2010). Species distribution and antimicrobial susceptibility of staphylococci isolated from canine otitis externa. Vet. Dermatol..

[17] De Martino L., Nocera F.P., Mallardo K., Nizza S., Masturzo E., Fiorito F., Iovane G., Catalanotti P. (2016). An update on microbiological causes of canine otitis externa in Campania Region, Italy. Asian Pac. J. Trop. Biomed..

[18] Rosales R.S., Ramírez A.S., Moya-Gil E., de la Fuente S.N., Suárez-Pérez A., Poveda J.B. (2024). Microbiological survey and evaluation of antimicrobial susceptibility patterns of microorganisms obtained from suspect cases of canine otitis externa in Gran Canaria, Spain. Animals.

[19] Tesin N., Stojanovic D., Stancic I., Kladar N., Ružić Z., Spasojevic J., Tomanic D., Kovacevic Z. (2023). Prevalence of the microbiological causes of canine otitis externa and the antibiotic susceptibility of the isolated bacterial strains. Pol. J. Vet. Sci..

[20] Bourély C., Cazeau G., Jarrige N., Leblond A., Madec J.Y., Haenni M., Gay E. (2019). Antimicrobial resistance patterns of bacteria isolated from dogs with otitis. Epidemiol. Infect..

[21] Scherer C.B., Botoni L.S., Coura F.M., Silva R.O., dos Santos R.D., Heinemann M.B., Costa-Val A.P. (2018). Frequency and antimicrobial susceptibility of *Staphylococcus pseudintermedius* in dogs with otitis externa. Ciência Rural.

[22] Rubin J.E., Ball K.R., Chirino-Trejo M. (2011). Antimicrobial susceptibility of *Staphylococcus aureus* and *Staphylococcus pseudintermedius* isolated from various animals. Can. Vet. J..

[23] Norström M., Sunde M., Tharaldsen H., Mørk T., Bergsjø B., Kruse H. (2009). Antimicrobial resistance in *Staphylococcus pseudintermedius* in the Norwegian dog population. Microb. Drug Resist..

[24] Bugden D.L. (2013). Identification and antibiotic susceptibility of bacterial isolates from dogs with otitis externa in Australia. Aust. Vet. J..

[25] European Medicines Agency (2022). Advice on Designation of Antimicrobials or Groups of Antimicrobials Reserved for Treatment of Certain Infections in Humans in Relation to Implementing Measures Under Article 37(5) of Regulation (EU) 2019/6 on Veterinary Medicinal Products. https://food.ec.europa.eu/system/files/2022-03/ah_vet-med_imp-reg-2019-06_ema-advice_art-37-5.pdf.

[26] Papić B., Golob M., Zdovc I., Kušar D., Avberšek J. (2021). Genomic insights into the emergence and spread of methicillin-resistant *Staphylococcus pseudintermedius* in veterinary clinics. Vet. Microbiol..

[27] Iorgoni V., Iancu I., Popa I., Gligor A., Orghici G., Sicoe B., Dreghiciu C., Purec D., Nistor P., Florea B. (2025). First Report of Respiratory Infection Caused by Multidrug-Resistant *Escherichia coli* in an Ostrich in Romania. Antibiotics.

[28] Kadlec K., Schwarz S. (2012). Antimicrobial resistance of *Staphylococcus pseudintermedius*. Vet. Dermatol..

[29] Rubin J.E., Chirino-Trejo M. (2011). Prevalence, sites of colonization, and antimicrobial resistance among *Staphylococcus pseudintermedius* isolated from healthy dogs in Saskatoon, Canada. J. Vet. Diagn. Investig..

[30] Cain C.L. (2013). Antimicrobial resistance in staphylococci in small animals. Vet. Clin. North America. Small Anim. Pract..

[31] Usui M., Sabala R.F., Morita S., Fukuda A., Tsuyuki Y., Torii K., Nakamura Y., Okamura K., Komatsu T., Sasaki J. (2025). Antimicrobial susceptibility and genetic diversity of staphylococcus pseudintermedius isolated from companion animals and human clinical patients in Japan: Potential zoonotic implications. J. Glob. Antimicrob. Resist..

[32] Srednik M.E., Perea C.A., Giacoboni G.I., Hicks J.A., Foxx C.L., Harris B., Schlater L.K. (2023). Genomic Features of Antimicrobial Resistance in *Staphylococcus pseudintermedius* Isolated from Dogs with Pyoderma in Argentina and the United States: A Comparative Study. Int. J. Mol. Sci..

[33] Schwarz S., Silley P., Simjee S., Woodford N., van Duijkeren E., Johnson A.P., Gaastra W. (2010). Assessing the antimicrobial susceptibility of bacteria obtained from animals. J. Antimicrob. Chemother..

[34] Văduva D.B., Velimirovici D.E., Vaduva M.M., Stanga L., Petrescu H.P., Rada M.P., Cipu D., Vaduva B.M., Rădulescu M. (2018). Phenotypic Study and Sensitivity to Anti-Infective Chemotherapy of Bacterial Strains Isolated from Cutaneous-Mucosal Infections. Mater. Plast..

[35] Nocera F.P., De Martino L. (2024). Methicillin-Resistant *Staphylococcus pseudintermedius*: Epidemiological Changes, Antibiotic Resistance, and Alternative Therapeutic Strategies. Vet. Res. Commun..

[36] Feuer L., Frenzer S.K., Merle R., Bäumer W., Lübke-Becker A., Klein B., Bartel A. (2024). Comparative Analysis of Methicillin-Resistant *Staphylococcus pseudintermedius* Prevalence and Resistance Patterns in Canine and Feline Clinical Samples: Insights from a Three-Year Study in Germany. Antibiotics.

[37] Tanveer M., Ntakiyisumba E., Hirwa F., Yoon H., Oh S.-I., Kim C., Kim M.H., Yoon J.-S., Won G. (2024). Prevalence of Bacterial Pathogens Isolated from Canines with Pyoderma and Otitis Externa in Korea: A Systematic Review and Meta-Analysis. Vet. Sci..

[38] Wang Q., Chen S., Ma S., Jiao Y., Hong H., Wang S., Huang W., An Q., Song Y., Dang X. (2025). Antimicrobial Resistance and Risk Factors of Canine Bacterial Skin Infections. Pathogens.

[39] Iorgoni V., Iancu I., Popa I., Gligor A., Orghici G., Sicoe B., Badea C., Dreghiciu C., Luca I., Nistor P. (2025). The First Report of a Pulmonary Abscess Due to *Streptococcus intermedius* in Rabbits in Romania. Microorganisms.

[40] Iancu I., Popa S.A., Degi J., Gligor A., Popa I., Iorgoni V., Nistor P., Imre K., Nichita I., Herman V. (2025). Aerobic uterine pathogens in dairy cattle: Surveillance and antimicrobial resistance profiles in postpartum endometritis. Antibiotics.

[41] Clinical and Laboratory Standards Institute (CLSI) (2018). Performance Standards for Antimicrobial Disk and Dilution Susceptibility Tests for Bacteria Isolated from Animals.

